# Green-Engineered Cementitious Composite Production with High-Strength Synthetic Fiber and Aggregate Replacement

**DOI:** 10.3390/ma15093047

**Published:** 2022-04-22

**Authors:** Chaoshu Fu, Mingzhao Chen, Rongxin Guo, Rongqing Qi

**Affiliations:** 1 Faculty of Civil Engineering and Mechanics, Kunming University of Science and Technology, Kunming 650504, China; fuchos@stu.kust.edu.cn (C.F.); 13056682443@163.com (M.C.); qrqing@swfu.edu.cn (R.Q.); 2 Yunnan Key Laboratory of Disaster Reduction in Civil Engineering, Kunming 650504, China; 3 College of Civil Engineering, Southwest Forestry University, Kunming 650224, China

**Keywords:** green lightweight engineered cementitious composites, high-strength polyethylene fibers, low-toughness matrix, fly ash cenospheres, costs, carbon emissions

## Abstract

Engineered cementitious composites (ECCs) are potentially useful structural reinforcement and repair materials. However, owing to their high costs and carbon emissions, they are not used extensively. To control these carbon emissions and costs, recycled fly ash cenospheres (FACs) and high-strength polyethylene (PE) fibers are used here to explore the possibility of developing green lightweight ECCs (GLECCs). A series of experiments was conducted to test the physical and mechanical properties of the developed GLECC and to evaluate the possibility of developing an GLECC. The crack width development of the GLECC was also analyzed using the digital image correlation method. The experimental results indicate the following: (1) The increase in FAC content and the decrease in PE content worsened the performance of GLECCs, but the resulting GLECCs still had significant strain-hardening properties; (2) The performance and costs of GLECCs can be balanced by adjusting the amount of FAC and PE. The maximum amount of FACs attainable is 0.45 (FAC/binder), and the required amount of PE fibers can be reduced to 1%. As a result, the cost was reduced by 40% and the carbon emission was reduced by 36%, while the compressive strength was greater than 30 MPa, the tensile strength was greater than 3.5 MPa, and the tensile strain was nearly 3%. (3) The width of the crack was positively correlated with the FAC content and negatively correlated with the fiber content. In the 0.8% strain range, the average crack width can be controlled to within 100 μm and the maximum crack width can be controlled to within 150 μm, with the performance still meeting the requirements of many applications.

## 1. Introduction

Ordinary concrete is prone to brittle cracking during its service life, which compromises the safety and durability of the structure. Adding various types of fibers to concrete somewhat improves its toughness [1,2,3] and alleviates the cracking problem. However, fiber-reinforced concrete exhibits strain softening after cracking, meaning the structural durability problem remains unsolved. Under uniaxial tensions, engineering cementitious composites (ECCs) exhibit strain hardening behaviors, which strain can reach 3–8%, which is 300–800 times greater than that of ordinary concrete and fiber-reinforced concrete. Additionally, ECCs exhibit multiple cracking behaviors under tensile loads, with a high crack control ability. The crack width in ECCs is usually less than 100 μm. The unique highly tensile ductility and small crack width of ECCs mean they can overcome many of the challenges related to brittleness and crack damage in ordinary concrete, thereby improving the quality and durability of the infrastructure significantly [4].

To maintain the characteristics of strain hardening and multiple cracking, a small amount of fine quartz sand (<300 μm) is used in ECCs. Therefore, compared with ordinary concrete, the amount of cement in ECCs is usually high, which results in higher costs and carbon emissions [5]. Additionally, expensive synthetic polymer fibers should be added to ECCs to maintain their high tensile strain capacity. The price of synthetic fibers is high, and they are difficult to obtain, which also increases the costs and carbon emissions related to ECCs. In response to climate and resource issues, several studies [6,7,8,9,10,11,12,13,14,15] have been carried out within the building materials industry to improve material mechanics and green properties, and they have yielded encouraging results. Many studies have also been carried out on the carbon emissions and costs of ECCs. Regarding the cementitious materials, slag [16], glass pozzolans [17], sugarcane bagasse ash [18], and limestone calcined clay blend [19] have been used to partially replace cement in the development of green ECC, and geopolymers [20] have also been developed to completely replace cement in the production of ECC. Given the cost of quartz sand, researchers have used recycled rubber [21], recycled asphalt [22], recycled fine aggregate [23], and sea sand [24] as fine aggregates to produce green ECCs. The mechanical properties of the obtained green ECC are fairly weak but improve durability. In addition, low-cost unoiled polyvinyl alcohol (PVA) [4,25] and high-strength polypropylene fibers [26], recycled PET [27,28], and recycled tire polymer fibers [29] have also been used to partially replace PVA fibers in the production of low-cost ECCs. The obtained material had a tensile strain capacity of 1–3%, which is significantly lower than that of conventional ECCs, but it can still satisfy many engineering applications. The above results demonstrate that the partial or complete replacement of the components of ECCs with low-cost materials and recycled waste may weaken the mechanical properties to a certain extent but reduces material costs and carbon emissions. However, according to the research of Yu [27] and Leung [30], for a typical ECC-M45, the cost of PVA accounts for 80% of the total cost of ECCs, and it is difficult to achieve a substantial reduction in ECC cost and carbon emissions using only fiber substitution and aggregate substitution.

According to the microscopic design principles of ECCs, high fiber bridging surplus energy and low matrix fracture toughness are beneficial to ECC multiple cracking. When the fiber bridging energy is greater than the matrix fracture toughness and has a certain surplus value, the stable multiple cracking of ECC can be realized. Therefore, when seeking ways to reduce the costs associated with ECC, this principle should also be observed. In recent years, high-tensile strength polyethylene (PE) fibers have been used to develop ECCs with ultra-high ductility (tensile strain of 8–12%) and ultra-high strength (compressive strength of 20–120 MPa) [31,32]. Owing to their hydrophobic nature, PE fibers have no chemical bonds with the mortar matrix, only relying on friction bonds, and PE fibers have a tensile strength of up to 2900 MPa, which is 1.8 times that of PVA fiber (1600 MPa). Therefore, when PE fibers are subjected to bridging stress, they can be easily degummed and pulled out instead of breaking up [33]. This characteristic of PE fibers is conducive to providing high complementary energy to ECCs. Therefore, ECCs prepared using PE fibers have high tensile ductility. However, not all engineering applications require such a high tensile strain capability. Under the premise of meeting engineering needs, we might reduce the content of PE fiber to balance the costs and ensure the mechanical properties of ECCs [34]. Recently, lightweight aggregates with a low density have been used to develop lightweight ECCs (LECCs) with a low density of 1200–1900 kg/m^3^ [35]. Because of their low specific gravity, LECCs have broad application prospects in bridges, high-rise buildings, and floating platforms that are weight-sensitive. The introduction of lightweight fillers ensures LECCs have lower matrix fracture toughness, which may be conducive to multiple cracking of ECC. Therefore, combining the high-strength properties of PE fibers and the low-toughness properties of LECCs may provide more space for balancing the number of ECC fibers with the cost and mechanical properties. Fly ash cenospheres (FACs) are an industrial waste produced by coal-fired power plants, and they have a composition similar to that of fly ash. They have a hollow spherical structure with a density of only 450–900 kg/m^3^. Using FACs as a lightweight filler of LECCs facilitates the balancing of the performance and costs of ECC, as well as the recycling of waste.

This paper proposes the combination of high-strength fibers and recycled wastes to achieve a balance of ECC performance and cost with carbon emissions. Due to the introduction of lightweight fillers, the interface performance of the fiber/matrix in the ECC will be weakened, thereby reducing the capacity for fiber bridging, which may lead to a mismatch between the fiber bridging performance and the matrix fracture toughness, and the ECC may lose its multiple cracking characteristics. Therefore, we have undertaken a series of experiments to evaluate the possibility of using low-content PE fibers and lightweight recycled FACs fillers to develop green lightweight engineered cementitious composites (GLECCs). The mechanical properties of the materials developed were tested, and their cost and environmental impacts were evaluated.

## 2. Material Design Criteria

The tensile strain hardening properties of ECC are related to its stable multiple cracking. When the ECC mortar matrix is stressed and reaches the cracking point, the matrix will crack. Here, the stress at the crack is carried by the fiber. The fibers rely on interfacial bonding with the matrix to transmit forces back to the surrounding uncracked matrix. As the stress elevates up to the cracking strength of the other parts of the matrix, new cracks will be formed, meaning new cracks are continuously formed, producing multiple cracking. To ensure multiple cracking, it is first necessary to ensure that the cracking stress of the matrix (*σ_fc_*) is less than the maximum strength of the fiber (*σ*_0_)—the so-called strength criterion—and the greater the maximum bridging strength, the greater the likelihood of multiple cracking [36].

(1) $$\sigma_{fc}<\sigma_{0}$$

Secondly, in order to protect the strain hardening properties of the ECC, the cracks must be stable. During cracking, the maximum opening displacement of the crack will not increase with its expansion, and the crack will be flat. To ensure the generation of flat cracks, the energy balance in the crack propagation process must be satisfied. According to Marshall and Cox [16], the steady-state cracking mode can be assessed using the fiber bridging stress–crack opening (*σ-δ*) curve. To ensure the stable expansion of the cracks, the fracture energy of the crack tip (*J_tip_*) should be less than the complementary energy of fiber bridging (*J_b_*^′^).

(2) $$J_{tip}\leq \sigma_{0}\delta_{0}-\int_{0}^{\delta_{0}}\sigma (\delta )\equiv {J_{b}}^{\prime }$$

where *δ*_0_ is the crack opening corresponding to *σ*_0_.

The *J_b_*^′^ in the energy criterion establishes a relationship with the fiber/matrix interaction. The fiber–matrix interfacial bond is weak, and the fibers are easily pulled out of the matrix, which reduces the *σ*_0_ in the σ-δ curve. If the fiber matrix bond is too strong, the fibers will break easily, making the crack narrower. Both of these factors will lead to a reduction in *J_b_*^′^, causing it to fall short of the requirements of steady-state cracking.

A single-crack tensile test was used to quantify the *σ-δ* curve; the specific test method will be given in Section 3.4. The parameters *σ*_0_ and *δ*_0_ in formula (2) can be obtained from the curve, while *σ_fc_* is yielded from the matrix tensile test. *J_tip_* can be calculated by Equation (3).

(3) $$J_{tip}=K_{m}{}^{2}/E_{m}$$

where *E_m_* is the modulus of elasticity, which can be derived from the matrix tensile test, and *Km* is the fracture toughness of the matrix, which can be determined via a three-point bending test of the notched matrix beam (test details are shown in the next section). Equation (4) is used to calculate and determine *K_m_*.

(4) $$K_{m}=\frac{1.5(F_{Q}+\frac{mg}{2}\times 10^{-2})\times 10^{-3}\cdot S\cdot a_{0}{}^{1/2}}{th^{2}}f(\alpha )$$

(5) $$f(\alpha )=\frac{1.99-\alpha (1-\alpha )(2.15-3.93\alpha +2.7\alpha^{2})}{(1+2\alpha ){\left(1-\alpha \right)}^{3/2}},\alpha =\frac{a_{0}}{h}$$

The meanings of parameters *F_Q_*, *m*, *g*, *S*, *a*_0_, *t*, *h*, and *f(α)* are described in [37].

The distribution of fibers and defects in the matrix is random. To achieve the stable multiple cracking of ECC, a certain margin of fiber bridging strength and energy is required. Kanda [38] proposed a strain hardening index *(PSH)* to estimate the strength and energy margins.

(6) $$PSH(Strength)=\sigma_{0}/\sigma_{fc}$$

(7) $$PSH(Energy)={J_{b}}^{\prime }/J_{tip}$$

## 3. Materials and Test Procedures

### 3.1. Materials and Mix Proportions

**Figure 1:**
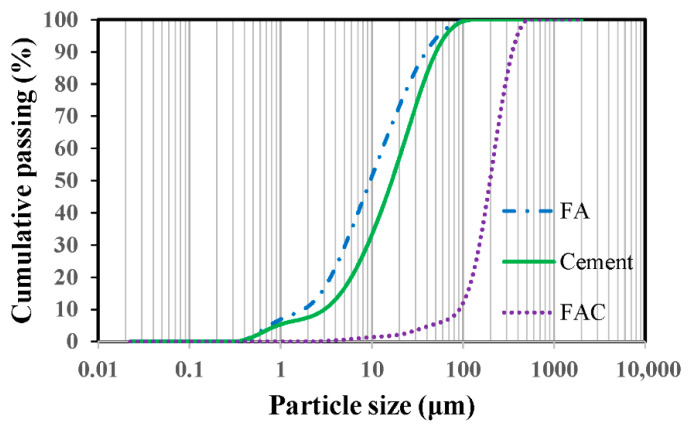
Particle size distributions of main raw materials.

**Figure 2:**
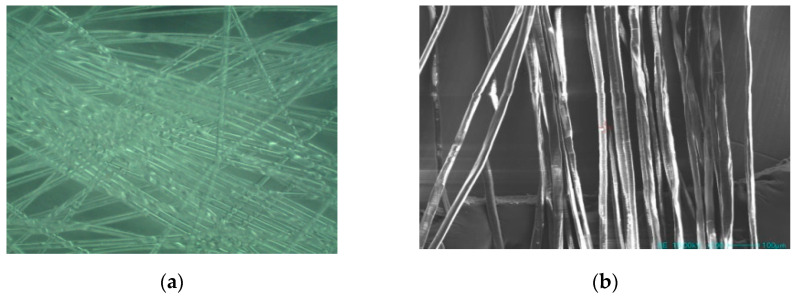
The microscopic images of PE fibers. (**a**) Optical microscope image; (**b**) SEM image (SE 1500 kV ×100).

Ordinary silicate P.O.52.5 grade cement was used as the main cementitious material, and F-type fly ash was used as the supplementary cementitious material. FACs, which are a kind of waste generated by coal-fired power plants, were used as fine aggregates. The FACs had a diameter of 0.01–0.5 mm and a density of 530 kg/m^3^. FACs can be used to obtain GLECCs with low fracture toughness and low density. Moreover, the waste can be recycled to improve the cost-efficiency and green performance. The chemical compositions of the main raw materials used for the preparation of the GLECC are listed in Table 1, and the particle size distribution is shown in Figure 1. Since the introduction of FACs will reduce the workability of GLECCs, a high-performance water-reducing agent (WR) was used in this study. A previous study [13] has indicated that PE fibers with high strength and a high elastic modulus can provide more space for balancing the tensile strain and volume fraction of GLECCs. Hence, PE fiber was used to prepare the GLECCs in this study. The physical and mechanical properties of PE fiber are listed in Table 2. Figure 2 shows the optical microscope and SEM images of the PE fibers.

Table 3 presents the GLECC mix proportions used in this study. First, by changing the FAC-to-binder mass ratio (FAC/binder: 0.15, 0.3, and 0.45), GLECC matrices were designed with three densities. Then, PE fibers of different volume fractions (*V_f_*) were added to the GLECC matrix to explore the feasibility of developing GLECCs with high FAC and low PE fiber contents. Regarding the high-density mixture (FAC/binder: 0.15), six fiber contents (*V_f_*: 1%, 1.25%, 1.5%, 1.75%, 2%, and 2.25%) were set to systematically study the effects of fiber content on the performance of GLECCs. For the low-density mixtures (FAC/binder: 0.3 and 0.45), only low fiber contents (*V_f_*: 1%, 1.25%, 1.5%) were added to verify the feasibility of developing GLECCs with low fiber contents and high FAC contents. Previous studies [39] have shown that the dispersion of fibers is closely related to the workability of the matrix. As the FAC content increased, the workability of the matrix reduced significantly. It is difficult to meet the requirements pertaining to fiber dispersion for the workability of the matrix by only changing the amount of WR. Therefore, as the content of FACs increased, a larger W/B was adopted. Additionally, at each density of GLECC, the proportion of WR was kept constant in order to study the effects of fiber content on the workability of the matrix. The nomenclature of the GLECCs is twofold: the first part designates the mass ratio of FACs (FAC/binder), whereas the second part designates the fiber volume fractions (*V_f_*). For example, the name of GLECC material FAC0.15−PE1 means that the mass ratio of FAC to binder is 0.15, and the fiber content is 1%.

### 3.2. Mixing, Casting and Curing Procedures

**Figure 3:**
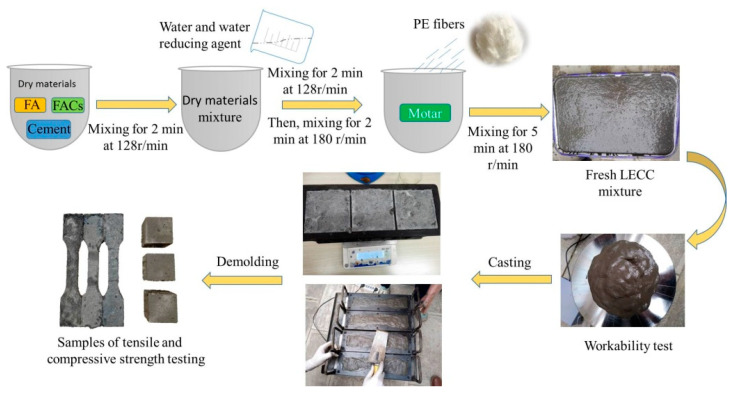
Sample preparation procedure.

The experimental procedures are shown in Figure 3, and the raw materials were weighed according to the mixing proportions shown in Table 3. The dry materials (including cement, FA, and FACs) were added to the planetary mixer and mixed at 128 r/min for 2 min. We then added the mixed solution of water and water reducing agent and continued to mix for 2 min, before mixing for 2 min at 180 r/min. Finally, the fibers were added and mixed at a 180 r/min mixing speed for 5 min to ensure their uniform distribution in the mortar. The fresh mixtures were cast into corresponding molds to be cured at room temperature and were demolded after one day. The specimens were cured to test age under standard curing conditions (temperature 20 ± 3 °C, relative humidity 95%) after demolding.

### 3.3. Workability of Mixture Test

After the mixtures were mixed, their flow diameters were tested via the method described in standard GB/T2419−2005 [40] to quantify the workability of the mixtures; the larger the flow diameter, the better the workability. First, fresh mixtures were poured into the molds. The mold was then removed from above, and the jumping table was turned on to vibrate the mixture at a frequency of 1 Hz for 25 s. The average values of the maximum diameter of the fresh mixture along the two vertical directions were calculated to obtain the spread diameters of the mixtures.

### 3.4. Strain Hardening Index Test

**Figure 4:**
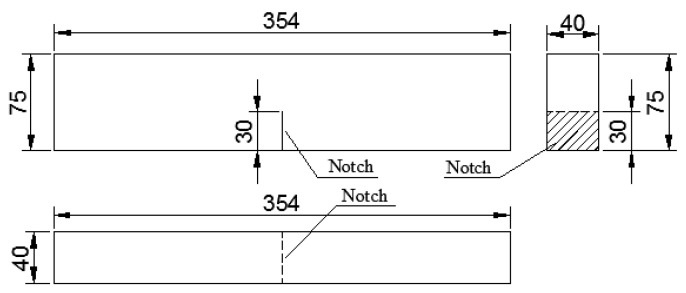
The size of the specimen tested for fracture toughness (Unit: mm).

**Figure 5:**
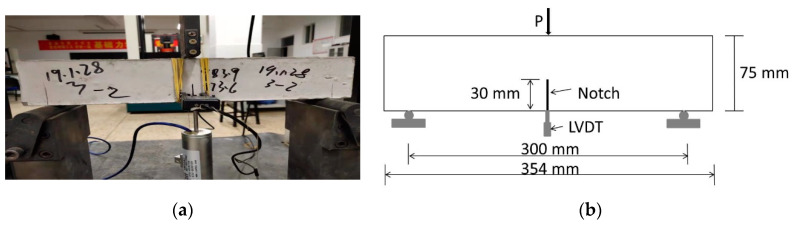
Fracture toughness test device. (**a**) Photo of the test device; (**b**) diagram of the test device.

The strain hardening index is an effective tool for predicting the multi-cracking probability of ECC. Three-point bending fracture toughness tests and single-crack tensile tests were used in this paper to quantify the probability of multi-cracking in GLECC. The specific experimental procedures are as follows:

**Fracture Toughness Test Method.** The fracture toughness of the GLECC matrix was tested with reference to the method of ASTM E399 [41], using a cuboid specimen of 354 × 75 × 40 mm^3^, as shown in Figure 4. Before the test, a notch 0.5 mm wide and 30 mm high was pre-cut into the middle of the specimen. The test was carried out with an electronic universal testing machine at a loading rate of 0.05 mm/min. During testing, a Linear Variable Differential Transformer (LVDT) was installed at the bottom of the specimen to measure its deformation, as shown in Figure 5.

**Figure 6:**
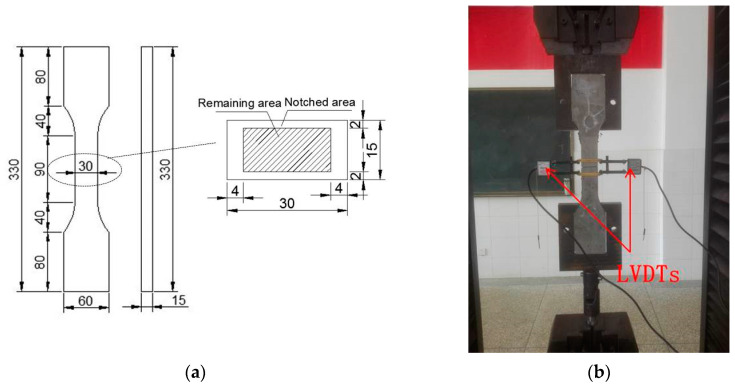
Single-crack tensile test. (**a**) Diagram of specimen size (Unit: mm); (**b**) photo of the test device.

**Test method of fiber-bridging complementary energy.** Via the method of [23,31], the fiber-bridging complementary energy was obtained using a single-crack tensile test. The dimensions of the sample are shown in Figure 6a. Before the test, an opening was pre-cut into the middle of the specimen (as shown in Figure 6a), the width of which was 0.5 mm. The test was carried out on a universal testing machine at a loading rate of 0.5 mm/min. During the test, two LVDTs were arranged near the opening to record its width, as shown in Figure 6b.

### 3.5. Physical and Mechanical Performance Test

**Bulk density**. The apparent density of the GLECC was tested on 50 × 50 × 50 mm^3^ cube specimens. After the samples were cured to the test age, the weight of the sample in the dry state of the surface was weighed, and three specimens were tested for each mix proportion.

**Compressive strength**. The specimens used in the compressive strength tests were the same as those used in the bulk density test. The compressive strength test was performed after the bulk density test. Here, a loading rate of 0.6 MPa/s was used, and three specimens were tested for each mix proportion [42].

**Figure 7:**
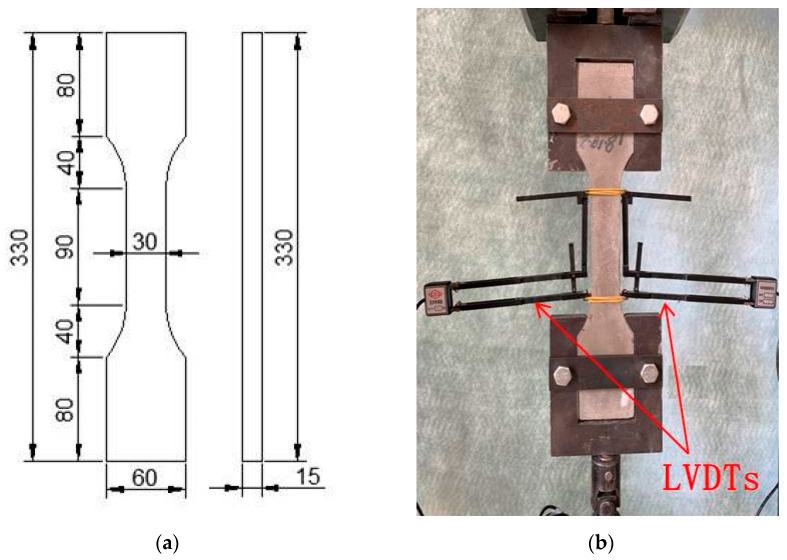
Uniaxial tensile test. (**a**) Diagram of specimen size (Unit: mm); (**b**) photo of the test device.

**Uniaxial tensile test**. Dog-bone specimens were used to test the tensile properties of the GLECC, and the dimensions of the specimens are shown in Figure 7a. The tensile test was carried out on a universal testing machine at a loading rate of 0.5 mm/min. During the tensile test, two LVDTs were fixed on either side of the specimen to record the deformation of the GLECC gauge length, as shown in Figure 7b.

### 3.6. Crack Characteristic Analysis

The tensile crack characteristics of ECCs are of broad concern. In this study, digital image correlation (DIC) technology was used to analyze the crack characteristics of GLECCs. A conceptual introduction of the DIC method can be found in References [43,44]. Before the tensile test, white and black paints were sprayed onto the surface of the sample to produce random speckled patterns such that each subset had a unique gray-scale distribution. During the tensile test, a CCD camera was installed on a tripod in front of the testing device. Digital images were captured every 5 s. These images are correlated to the actual tensile strain values via the time recording.

**Figure 8:**
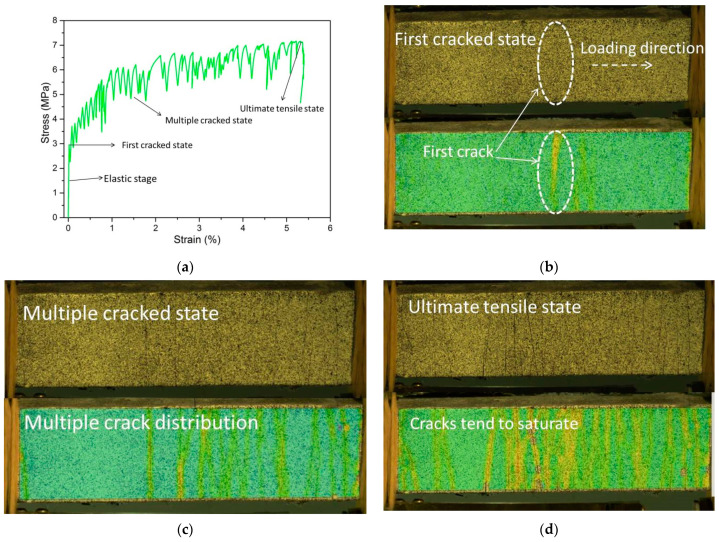
Strain field of GLECCs under different strains. (**a**) Tensile stress–strain curve of FAC0.15−PE1.5; (**b**) the strain contours of the initial crack state; (**c**) the strain contours of the multiple cracked state; (**d**) the strain contours of the ultimate tensile state.

After the image was captured, the software GOM Correlate was used to calculate the deformation field and strain field in the plane. Based on the calculated results of DIC, crack development in GLECCs with different mix proportions can be analyzed. Taking the FAC0.15−PE1.5 GLECCs as an example, we assess the strain contours at different stages (initial crack state, multi-crack crack state, and ultimate state) on the stress–strain curve, as shown in Figure 8. Because the strain value of the cracked area is much greater than that of the uncracked area, a crack can easily be identified by the corresponding strain profile. As shown in Figure 8b–d, it is difficult to identify a crack from the original picture of the tensile specimen, but cracks are easily captured in the pictures of the strain field distribution calculated by DIC.

**Figure 9:**
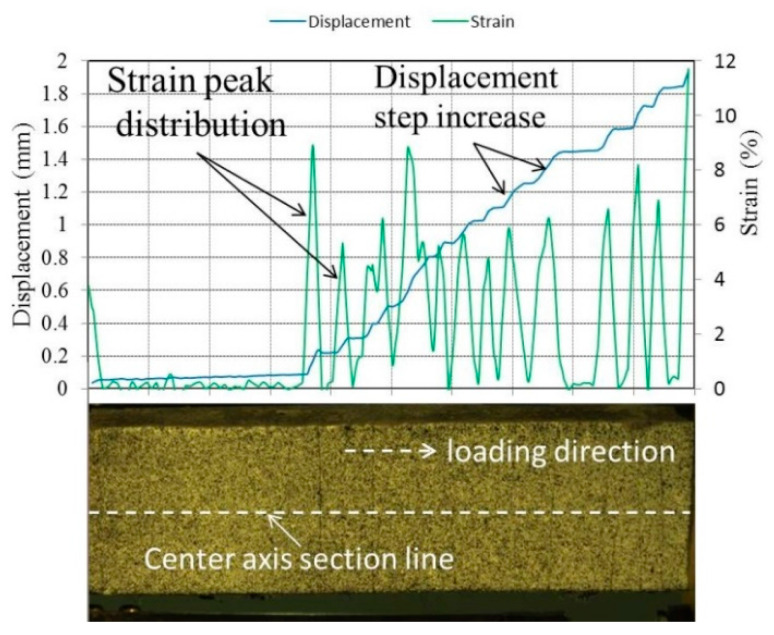
Displacement distribution and strain distribution of the central section.

After obtaining the corresponding displacement and strain fields, strain change at the cracking position can be observed according to the strain cloud diagram, and the strain change in the tensile direction can be used to infer the crack width. Figure 9 shows the displacement distribution and the strain distribution in the tensile direction, extracted from the central axis of the strain cloud of the specimen. When a crack occurs, the displacement in the loading direction will change in steps, while the strain will peak.

**Figure 10:**
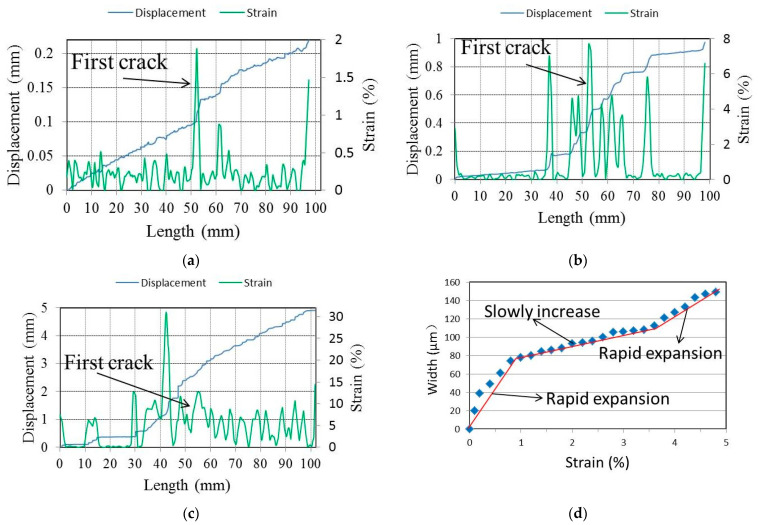
The strain and displacement distribution diagrams of the tensile specimen. (**a**) The initial crack state; (**b**) the multiple crack state; (**c**) the ultimate crack state; (**d**) the variation in the first crack’s width.

The crack distribution and propagation behavior of the specimen can be monitored according to the strain and displacement distribution diagrams at different stages. Figure 10a–c show the strain and displacement distribution diagrams of the specimen in the initial crack state, the multiple crack state, and the ultimate crack state, respectively. When the applied stress reaches the cracking strength of the specimen, a crack propagates from the middle of the specimen. With the further increase in load, multiple cracks form on the specimen. When approaching failure, the cracks tend to spread across the gauge length of the specimen, as shown in Figure 10c. In addition, changes in crack propagation behavior at different stages can be analyzed according to the deformation distribution. Taking the first crack as an example, the variation in crack width at each stage can be obtained according to the displacement distribution diagram, as shown in Figure 10d. The crack width increased rapidly at the initial stage of loading. When reaching a certain width, the increase gradually slowed, demonstrating the excellent crack control ability of ECC. The crack width increased rapidly until failure occurred. In this paper, this method will be used to analyze the crack behavior of GLECCs with different mix proportions.

## 4. Results and Discussion

### 4.1. Workability

**Figure 11:**
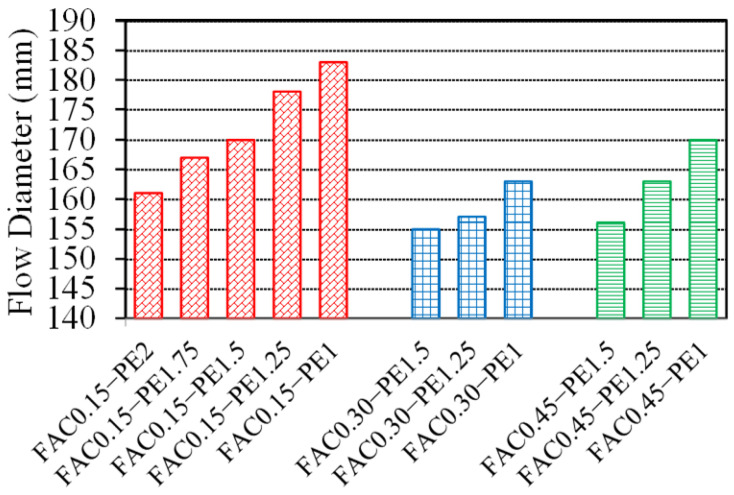
Flow diameters of GLECC mixtures.

Figure 11 shows the flow diameters of the GLECC mixtures. The fiber content had a significant impact on the workability of the GLECC mixtures. For any density of the GLECC mixtures, as the fiber content decreased, the flow diameter showed an increasing trend. This is because the randomly distributed fibers form a spatial network structure, which restricts the free flow of the fresh mixture. Thus, the higher the fiber content, the worse the workability of the mixture.

### 4.2. Bulk Density and Compressive Property

**Figure 12:**
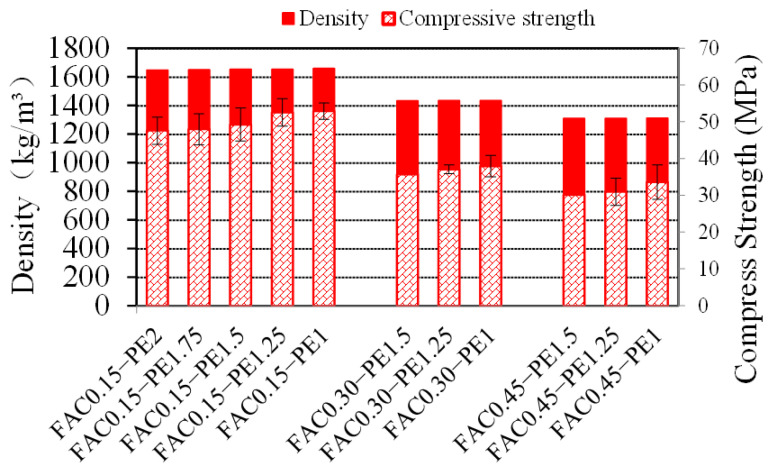
Summary of density and compression of GLECCs.

The bulk density and compressive properties are shown in Figure 12. FACs were used as lightweight fillers with a hollow structure, the density of which was only 530 kg/m^3^, while the density of cement was 3180 kg/m^3^. For a unit volume of GLECCs, the addition of more FACs will reduce the amount of cement used. Therefore, adding FACs to GLECCs can significantly reduce the density of the GLECCs. The density of FAC0.45 series GLECCs was 1310 kg/m^3^, which is 20% lower than that of the FAC0.15 series GLECCs. Concrete with a density of less than 1950 kg/m^3^ can be defined as lightweight concrete [45]. The GLECCs developed in this study had densities between 1310 and 1650 kg/m^3^, and they can all be defined as lightweight. Inevitably, the increase in FAC content deteriorated the mechanical properties of the GLECCs. With the increase in FAC proportion, the compressive strength showed a decreasing trend. However, the compressive strengths of all mixtures remained greater than 30 MPa. For any density of the GLECC mixtures, with the decrease in PE fiber content, the compressive strength showed an increasing trend. For example, the compressive strength of FAC0.15−PE1 was 52.8 MPa, which was approximately 10% higher than the 47.5 MPa of FAC0.15−PE2. This is because an increase in fiber content results in a decrease in the compactness of the matrix [46]. The incorporation of fibers introduces bubbles in the mixture. The higher the fiber content, the greater the number of bubbles introduced. Moreover, the bubbles were difficult to eliminate. These bubbles persist in the material as defects after the cement hardens. Furthermore, as the fiber content increased, fiber entanglement occurred during the mixing process. The greater the fiber content, the greater the number of fiber clusters. The entanglement of fiber clusters not only precludes an enhancement of crack resistance, but these clusters also exist as defects in the matrix, thereby affecting the compressive strength.

### 4.3. Strain Hardening Index

**Figure 13:**
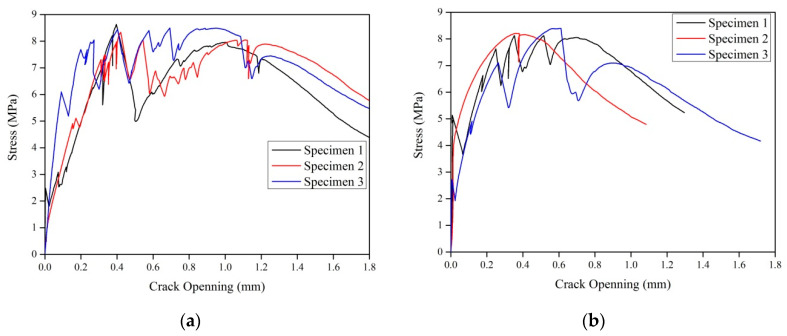

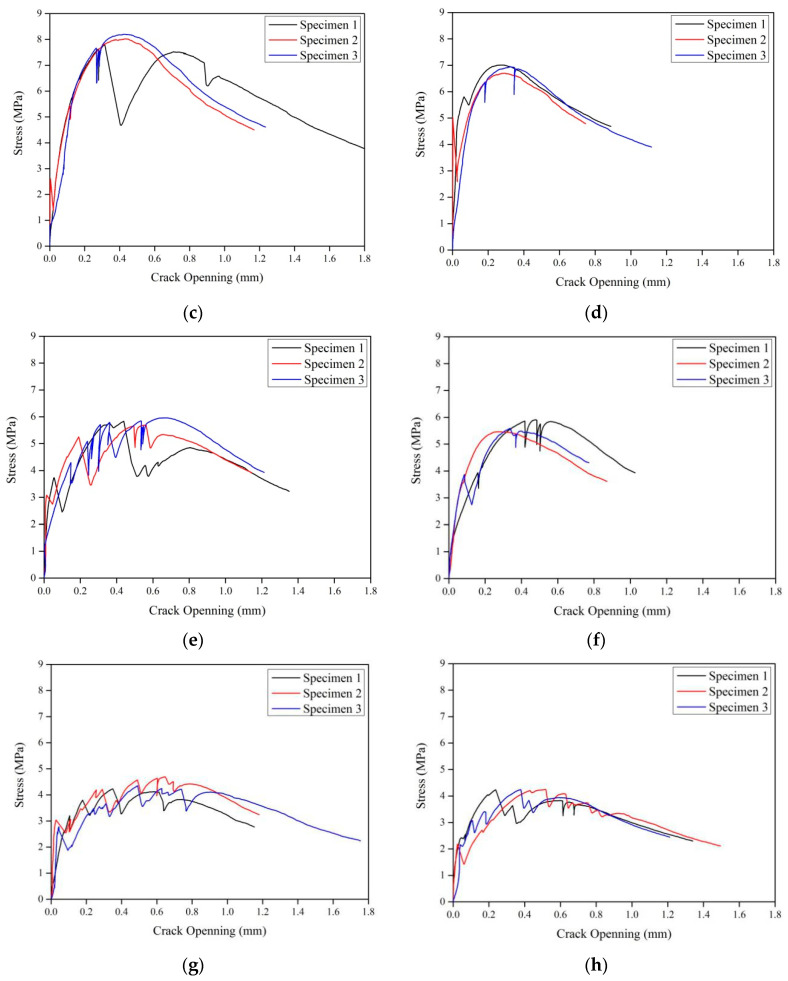
Fiber bridging stress–crack opening curves of GLECC mixtures. (**a**) FAC0.15−PE1.75; (**b**) FAC0.15−PE1.5; (**c**) FAC0.15−PE1.25; (**d**) FAC0.15−PE1; (**e**) FAC0.3−PE1.5; (**f**) FAC0.3−PE1; (**g**) FAC0.45−PE1.25; (**h**) FAC0.45−PE1.

As introduced in Section 2, the strain hardening index can be used to evaluate the possibility of multiple cracking in ECC. In this study, the fracture toughness of three GLECC matrices with different FAC contents was obtained via a three-point bending test performed on notched beams to analyze the effects of lightweight fillers on the matrix fracture toughness of GLECCs. Table 4 presents the test results. As expected, the introduction of lightweight fillers significantly reduced the fracture toughness of the GLECCs matrix. With the increase in FAC content, fracture toughness and fracture energy decreased. Low fracture toughness is beneficial to the realization of multiple cracking in GLECCs [36]. The bridging stress–crack opening curves obtained from the single-crack tensile test are presented in Figure 13. The *σ_0_* and *δ*_0_ values can be obtained from the curve, and the *J_b_*^′^ value of GLECCs can be calculated using Equation (2). The results are listed in Table 4. *σ_0_* and *J_b_*^′^ reflect the crack-bridging performance achieved by the fiber. With the same fiber content, as the FAC content increased, the *σ_0_* and *J_b_*^′^ both decreased, which indicates that the introduction of lightweight fillers weakened the fiber’s bridging performance. This is because, as the FAC content increased, the proportion of cementitious material in the mixture decreased, which means that more FACs were present around the fibers, weakening the fiber bridging performance [37]. As the fiber content decreased, *σ_0_* and *J_b_*^′^ decreased. For example, for a GLECC with an FAC content of 0.15, with the reduction in fiber content from 2% to 1%, the *σ_0_* was reduced from 9.43 MPa to 6.88 MPa (25.8%), and the *J_b_*^′^ was reduced from 1295.6 to 380.1 J/m^2^, a 70% reduction.

According to the above results, the introduction of lightweight fillers reduced the fracture toughness of the matrix while also weakening the fiber bridging performance, which is unfavorable to the balancing of fiber dosage and mechanical properties because a reduction in fiber dosage will also limit the fiber bridging performance. The degradation of fiber bridging performance caused by the combination of lightweight fillers and reduced fiber usage may prevent the strain hardening criterion from being met. We sought to comprehensively evaluate the possibility of GLECC multiple cracking when the amount of lightweight filler is increased and the amount of fiber is decreased. Based on the experimental results of fracture toughness and fiber bridging performance, the strain hardening index (*PSH (strength)* and *PSH (energy)*) were calculated using Equations (6) and (7). The results are listed in Table 4. As shown in Table 4, in the GLECC mixture with the same FAC content, with the decrease in fiber dosage, the *PSH (strength)* and *PSH (energy)* were greatly reduced, showing that fiber dosage had a significant effect on the stable multiple cracking of GLECCs. With the same value of fiber dosage, the increase in the FAC content had no obvious effect on *PSH (strength)* and caused the *PSH (energy)* to slightly increase. In general, reducing the amount of fiber and increasing the content of FAC will lead to a decrease in fiber bridging performance. However, thanks to the high strain hardening index surplus of high-strength PE fiber and the reduction in fracture toughness, the obtained material still had a large strain hardening index surplus value, indicating that the obtained material can achieve multiple cracking. For example, in the mixture FAC0.45−PE1, although the fiber content was reduced to 1%, it still had *PSH (strength)* and *PSH (energy)* values of 1.84 and 33.1. The evaluation of the strain hardening index suggests that it is feasible to develop a GLECC with a low content of high-strength PE fiber and a low-toughness matrix. The obtained material meets the strength and energy criteria and can realize multiple cracking, which is significant to balancing costs and mechanical properties.

### 4.4. Tensile Performance

**Figure 14:**
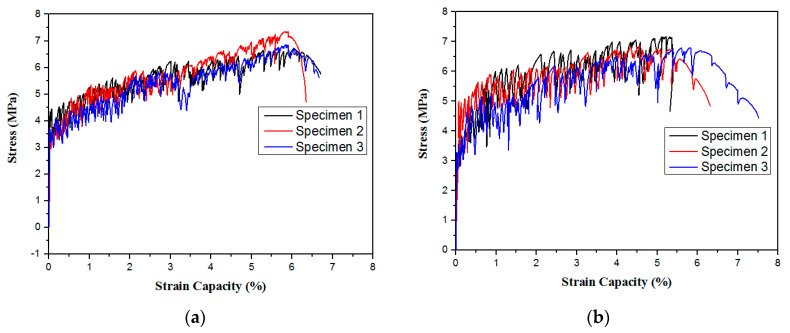

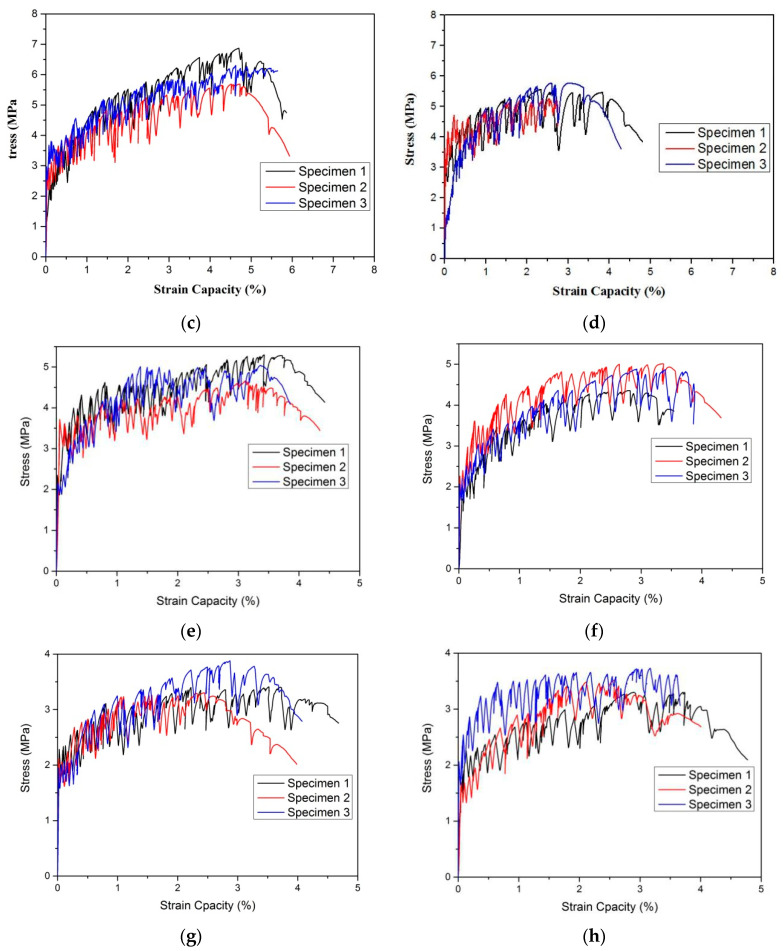
Tensile stress–strain curves of GLECC mixtures. (**a**) FAC0.15−PE1.75; (**b**) FAC0.15−PE1.5; (**c**) FAC0.15−PE1.25; (**d**) FAC0.15−PE1; (**e**) FAC0.3−PE1.5; (**f**) FAC0.3−PE1; (**g**) FAC0.45−PE1.25; (**h**) FAC0.45−PE1.

**Figure 15:**
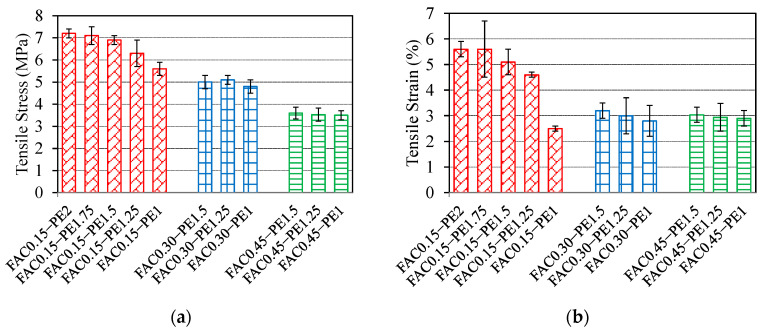
Tensile performances of GLECC mixtures. (**a**) Tensile stress; (**b**) tensile strain.

Figure 14 shows a tensile stress–strain relationship diagram for some GLECCs. The shapes of the GLECC stress–strain relationships for different fiber contents and FAC dosages are similar, and both contain an elastic section showing an increase in linear stress and a strain-hardening section with stress oscillation. However, the strength, strain, and stress shock amplitude exhibited were quite different. Decreases in fiber dosage and the increases in FAC proportion will weaken the tensile performances of GLECC, and a decrease in fiber dosage will increase the amplitude of stress shock. Figure 15 further compares the influence of fiber dosage and FAC proportion on the tensile performances of GLECCs. Ultimate tensile strength and ultimate tensile strain are useful parameters for evaluating the tensile properties of GLECCs. The ultimate tensile stress is obtained by dividing the maximum load that the specimen can carry throughout the entire tensile process by the cross-sectional area of the specimen. The strain of the specimen at the maximum stress is the ultimate tensile strain.

As shown in Figure 15, as the FAC proportion increased, the ultimate tensile strength and ultimate tensile strain decreased for GLECCs with the same fiber content. The ultimate tensile strength and ultimate tensile strain are related to the bridging performance of the fiber. An increase in the proportion of FAC weakens the confinement effect of the matrix on the fibers, resulting in the weakening of the fiber’s bridging ability and finally the deterioration of the tensile properties of GLECCs. The fiber content had a significant effect on the tensile performances of GLECCs. When the FAC content was constant, as the fiber content decreased, the tensile properties decreased. We take GLECCs with an FAC proportion of 0.15 as an example. As the fiber content decreased from 2% to 1%, the ultimate tensile strength and tensile strain decreased from 7.2 MPa and 5.6% to 5.6 MPa and 2.5%, i.e., a decrease of 20% and 50%, respectively. This is because the decrease in fiber content was accompanied by an attenuation of the fiber bridging performance, which caused a reduction in tensile properties [34]. However, due to the high bridging energy surplus value of PE fiber and the contribution of FAC to the reduction in the fracture toughness of the matrix, although the fiber content was reduced to 1%, the resulting material still had excellent tensile properties, with a tensile strain of nearly 3%, which is comparable to conventional ECC-M45.

The results of the above-mentioned tensile properties indicate that a reduction in fiber dosage can be achieved with high-strength PE fibers and a low fracture toughness matrix, which is of great significance for balancing the cost and tensile ductility of GLECCs. The developed GLECC mixtures still exhibit tensile strengths of up to 3.5–7.5 MPa and tensile strains of 2.5–5.5%, which values are close to those of the conventional ECC-M45, accompanied by densities as low as 1300–1650 kg/m^3^. As the tensile strain of an ordinary building during its normal service life rarely exceeds 3%, it is believed that the GLECCs we developed with low PE fiber contents and high FAC contents can still meet the requirements of ordinary building applications.

### 4.5. Analysis of Crack Characteristics

#### 4.5.1. Cracking Pattern

**Figure 16:**
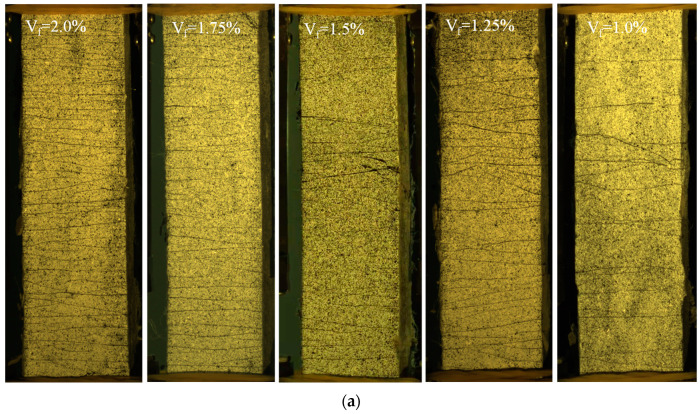

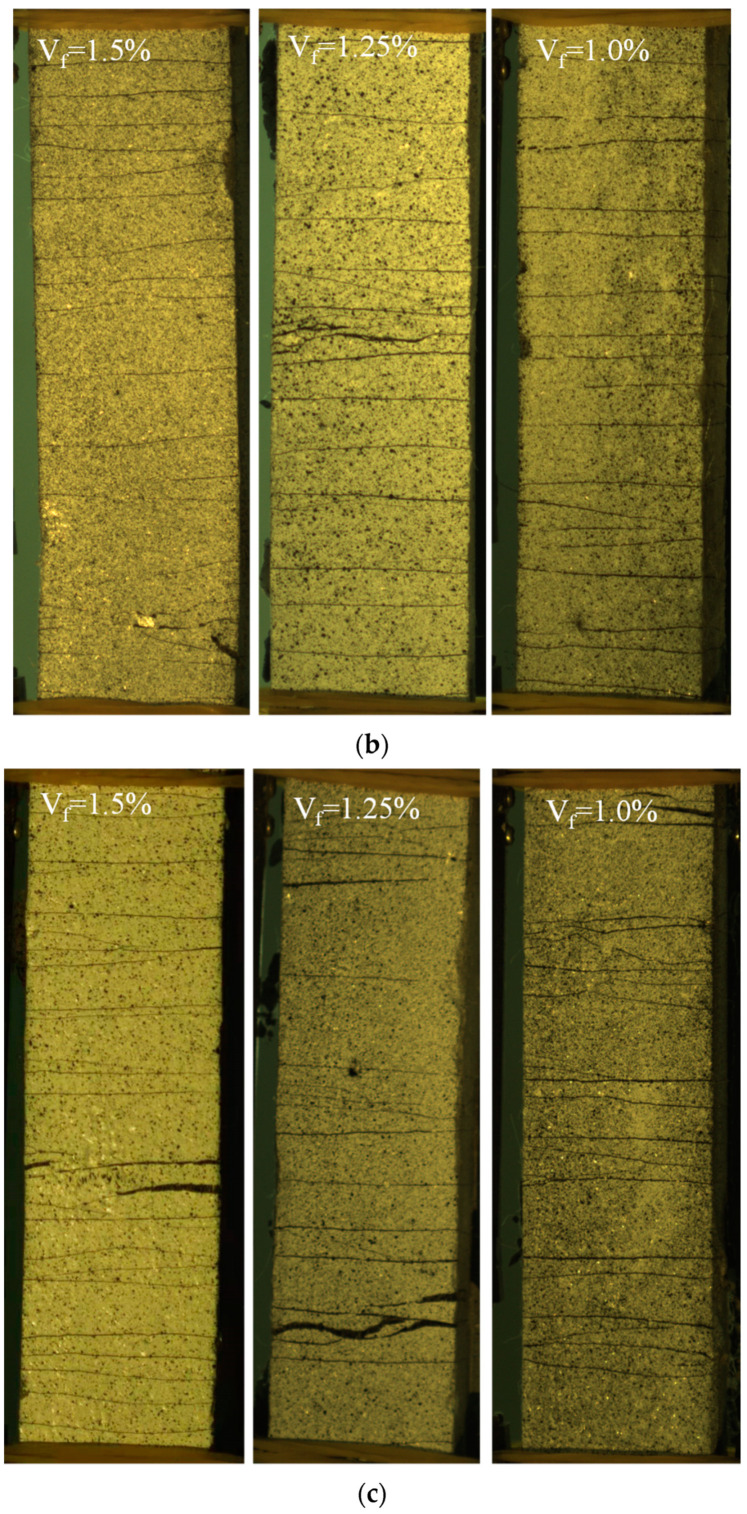
Cracking pattern of GLECC mixtures. (**a**) GLECCs with FAC content of 0.15; (**b**) GLECCs with FAC content of 0.3; (**c**) GLECCs with FAC content of 0.45.

Figure 16 shows the final crack patterns of GLECC tensile specimens with different fiber contents at three different densities. All of the GLECC specimens exhibited the multiple cracking behavior. The fiber content affected the crack pattern significantly. When the fiber contents were 2% and 1.75%, the cracks were denser, finer, and saturated in the target area, while under high tensile strain, no crack localization was observed, indicating that the fiber content of GLECCs saturated with multiple cracks was 1.75%. As the amount of fiber decreased, the cracks gradually become more sparse and wide. When the fiber content was 1%, the crack spacing reached 50–70 mm, constituting an unsaturated state.

#### 4.5.2. Crack Width Development Behavior

**Figure 17:**
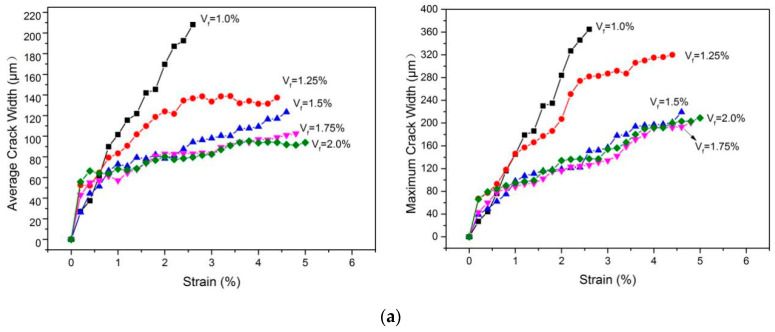

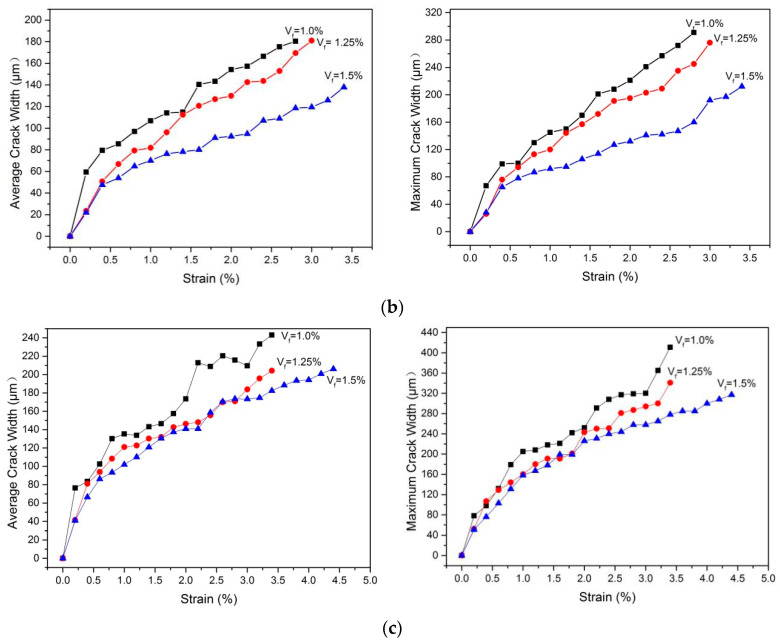
Crack development of GLECC mixtures containing different FAC content. (**a**) GLECCs with FAC content of 0.15; (**b**) GLECCs with FAC content of 0.3; (**c**) GLECCs with FAC content of 0.45.

The water permeability and self-healing ability of concrete are directly related to crack width. Studies [47,48,49,50] have shown that when the crack width is less than 100 μm, the water permeability and chloride ion diffusivity of concrete can be ignored. Moreover, concrete with a crack width of less than 150 μm also has a self-healing effect. Therefore, studying the relationship between the crack width and strain of GLECCs is of great significance when considering durability. Figure 17 shows the variation in the average and maximum crack widths of GLECC tensile specimens determined using the DIC method with increasing strain. The relationship curve between the average crack width and the deformation can yield cracking information for the material at a given strain, and the relationship curve between the maximum crack width and the strain provides the worst-case information [51]. As shown in Figure 17, the fiber content has a significant effect on the crack control ability. For GLECCs of the same density, with a decrease in fiber content, the average and maximum crack widths showed an increasing trend. For GLECCs with the same fiber content, the larger the FAC content, the worse the crack control ability. Furthermore, the maximum and average crack widths increased with increasing FAC proportions. This is because, in GLECCs with a high proportion of FACs, more FACs will be present around the fibers, thereby weakening the binding effect of the matrix with the fibers. Simultaneously, due to the addition of more FACs, the rheology of GLECCs will deteriorate, and more water will need to be added to meet the rheological requirements of fiber dispersion, resulting in a loss of strength. These two factors lead to a deterioration in fiber crack resistance.

In GLECCs with a relatively high density (FAC/binder: 0.15), when the PE content was greater than 1.5%, the GLECCs had a strong crack control ability; i.e., for FAC1.5−PE1.75 and FAC1.5−PE2, the average crack width could be kept below 100 μm, whereas the maximum crack width was below 200 μm (as shown in Figure 17a). However, when the fiber content decreased, the crack width control ability decreased, thereby increasing the crack width; i.e., for GLECC mixtures with FAC/binder: 0.15, under the same strain level, as the fiber content decreased, the average crack width and the maximum crack width both increased (as shown in Figure 17a). Moreover, for FAC1.5−PE1, FAC1.5−PE1.25, and FAC1.5−PE1.5, with 1.0% tensile strain, the average crack width was less than 100 μm, whereas the maximum crack width was less than 150 μm. As shown in Figure 17b,c, for low-density GLECCs (FAC/binder: 0.3 and 0.45), in the 0.8% strain range, the average and maximum crack widths were less than 100 μm and 150 μm, respectively. This shows that although reducing the amount of fiber increases the crack width of GLECCs, their durability will not be affected within a certain strain range. For low-density GLECCs, when the fiber content was less than 1.5%, the durability was not affected in the strain range of 0.8%. However, for relatively high-density GLECCs (FAC/B: 0.15), the strain can be relaxed to 1%. It is believed that strain values of 0.8% and 1% can still meet the needs of many applications, because structures rarely exceed 1% tensile strain during their normal service life. In order to meet the requirements of an ordinary structure under normal use conditions, the fiber content in GLECCs can be reduced to 1–1.5%.

### 4.6. Cost and Carbon Emission Analysis

**Figure 18:**
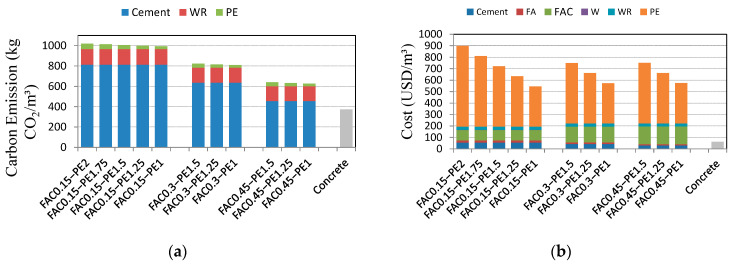
Cost and CO_2_ emissions of GLECCs. (**a**) Carbon emission; (**b**) cost [28,52,53,54].

To evaluate the green performance of GLECCs, their costs and CO_2_ emissions were calculated. The costs and CO_2_ emissions of the main raw materials used to produce GLECCs are listed in Table 5. The data were collected from the published literature and various material suppliers. Since FACs and FA are wastes, CO_2_ emissions were not generated by default in the calculation. According to the quantity, cost, and CO_2_ emissions of the raw materials listed in Table 3 and Table 5, the cost and CO_2_ emission index of producing a unit volume of GLECCs can be calculated. The results are shown in Figure 18. As a comparison, this study also outlines the cost and carbon emissions of ordinary concrete. The carbon emissions and costs of GLECCs were much higher than those of ordinary concrete. Owing to the application of more cement and fiber, the carbon emissions were mainly derived from cement, whereas the increase in cost was mainly derived from fiber. Therefore, as expected, increasing the use of FACs can significantly reduce the amount of cement, thereby reducing carbon emissions. Specifically, when the fiber content was constant, increasing the FAC content from 0.15 to 0.45 could result in the carbon emissions of GLECCs being reduced by 36%. Reducing the amount of fiber can significantly reduce the costs of the materials. For example, for GLECCs with an FAC content of 0.15, as the fiber content was reduced from 2% to 1%, the cost was reduced by approximately 40%. Increasing the FAC content and reducing the fiber content deteriorated the mechanical properties, but the developed GLECCs still had an ultimate tensile strain of approximately 3% and a tensile strength of more than 3.5 MPa, and the compressive strength was 30–50 MPa. For the standard ECC-M45, the compressive strength is 40 MPa, the tensile strength is 4.5 MPa, and the tensile strain is 3% [52]. Therefore, the performance of the GLECCs developed in this study is comparable to that of the standard ECC-M45, illustrating the availability of GLECCs. Moreover, in the 0.8% tensile strain range, the average and maximum crack widths of GLECCs were less than 100 μm and 150 μm, respectively. Since the penetration rate of water and chloride ions is very small when the crack width is less than 100 μm, it can be ignored in engineering [47,48]. In other words, the durability of GLECCs was not affected when the strain did not exceed 0.8%. The tensile strain of concrete is only 0.01%. GLECCs can function effectively in many scenarios in which concrete is ineffective. It can be concluded that GLECCs can meet many engineering needs, with significantly lower costs and carbon footprints.

## 5. Conclusions

This study has aimed to explore the feasibility of using high-strength PE fibers and recycling FACs to develop GLECCs. The results show that by adjusting the FAC and PE contents in GLECCs, the production cost and carbon emissions, which are of practical significance when balancing the performance and costs of GLECCs, can be reduced considerably. The following conclusions can be drawn based on the results of this study.

(1) FACs were effective in reducing the density of GLECCs. However, they also weakened the mechanical properties of GLECCs. With an increase in the FAC content, the compressive and tensile strengths showed a decreasing trend. However, the obtained compressive and tensile strengths of GLECCs were still greater than 30 MPa and 3.5 MPa, respectively.
(2) The PE fiber affects the workability of GLECC adversely. As the fiber content increased, the flow diameter showed a decreasing trend. Additionally, it was observed that as the fiber content increased, the compressive strength of GLECCs showed a decreasing trend. In GLECCs with an FAC content of 0.15, the compressive strength of FAC0.15−PE2.25 was 44.2 MPa, which is approximately 16% lower than the 52.8 MPa of FAC0.15−PE1.
(3) Reducing the amount of fiber will result in a decrease in the fiber’s bridging performance, resulting in a decrease in the tensile properties. All GLECCs exhibited obvious strain hardening, with a tensile strength of 3.5–7.5 MPa and a tensile strain of 2.5–5.5%. In GLECCs of any density, even when the fiber content was as low as 1%, the strain hardening behavior was still obvious. The tensile strain was close to 3%, whereas the tensile strength was greater than 3.5 MPa.
(4) The width of the crack is positively correlated with the FAC content and negatively correlated with the fiber content. Although a change in FAC and fiber dosage resulted in a change in the crack width, the durability of GLECCs was not affected within a certain strain range. Even when the fiber content was as low as 1%, in the 0.8% strain range, the average crack width and maximum crack width remained at 100 μm and 150 μm, respectively, thus meeting the durability requirements of conventional applications.
(5) The use of recycled FACs and PE can significantly reduce the cost and environmental impact of GLECCs. As the FAC content increased from 0.15 to 0.45, the carbon emissions of GLECCs were reduced by as much as 36%, and as the fiber content was reduced from 2% to 1%, the cost was reduced by approximately 40%. Nevertheless, the performances of the GLECCs are sufficient for many applications.

This study provides a reference for the design and utilization of GLECCs. Crack width only offers a qualitative insight into durability. Further research should be undertaken to further evaluate the durability of GLECCs, including their resistance to carbonization, the chloride ion penetration, and the sulfate attack resistance. The width of the crack has a significant effect on the self-healing performance of GLECCs, which should be further evaluated quantitatively. In addition, FACs has certain pozzolanic capacities, the impacts of which on the long-term performance of GLECCs should be further considered.

## Figures and Tables

**Table 1:** Chemical composition and physical properties of main raw materials.

Chemical Analysis	Cement (wt. %)	FA (wt. %)	FACs (wt. %)
CaO	65.41	3.3	1.06
Fe_2_O_3_	3.2	8.09	1.96
MgO	1.58	1.34	-
SO_3_	5.72	0.67	0.42
K_2_O	0.5	1.37	3.94
SiO_2_	19.47	53	73.1
Na_2_O	-	0.34	2.42
Al_2_O_3_	3.86	24.19	16.7
TiO_2_	0.26	-	0.35
Others	-	7.7	0.05
Particle size	15 µm	10 µm	0.01–0.5 mm
Specific gravity (g/cm^3^)	3.18	2.68	0.53
Specific surface area (m^2^/g)	0.438	0.663	-

**Table 2:** Physical and mechanical performances of PE fibers.

	PE Fiber
Length/mm	12
Aspect ratio	460
Tensile strength/GPa	2.9
Young’s modulus/GPa	116
Density/g/cm^3^	0.97

**Table 3:** Mix proportions of GLECCs.

Mix ID	Cement (kg/m^3^)	FA (kg/m^3^)	FACs (kg/m^3^)	Water (kg/m^3^)	WR (kg/m^3^)	PE (*V_f_*) (%)
FAC0.15−PEn	913	391.5	195.8	251.7	101.7	*n*
FAC0.30−PEn	685.1	293.6	293.6	225	87.5	*n*
FAC0.45−PEn	511.9	219.4	330	213.3	86.6	*n*

For the GLECCs with an FAC content of 0.15, *n* = 1, 1.25, 1.5, 1.75 and 2. For the GLECCs with FAC contents of 0.3 and 0.45, *n* = 1, 1.25, and 1.5.

**Table 4:** Strain hardening index analysis of GLECCs.

Mix ID	*F_Q_* (N)	*K_m_* (MPa·m^1/2^)	*J_tip_* (J/m^2^)	*σ*_0_ (MPa)	*PSH (Strength)*	*J_b_*^′^ (J/m^2^)	*PSH (Energy)*
FAC0.15−PE2	751.7	0.6	18.63	9.43	2.48	1295.6	66.6
FAC0.15−PE1.75				8.48	2.23	1280.9	65.9
FAC0.15−PE1.5				8.25	2.17	930.8	47.9
FAC0.15−PE1.25				7.57	1.99	777.1	39.9
FAC0.15−PE1				6.88	1.81	380.1	19.5
FAC0.3−PE1.5	576.4	0.46	15.35	5.9	1.9	827.8	49.8
FAC0.3−PE1.25				5.82	1.88	703.2	42.3
FAC0.3−PE1				5.65	1.82	581.8	35
FAC0.45−PE1.5	481	0.39	13.85	4.44	1.96	594.8	44.9
FAC0.45−PE1.25				4.42	1.92	557.4	42.1
FAC0.45−PE1				4.24	1.84	438.9	33.1

**Table 5:** Costs and CO_2_ emissions of ECC raw materials.

Chemical Analysis	Cost (USD/t)	CO_2_ Emissions (kg/m^3^)
Cement	62.6	930 [28,53]
FA	46.9	-
FACs	469	-
W	0.9	-
WR	312	1667 [54]
PE	35200	2671
Concrete	61.5	373 [52]

## Data Availability

The data presented in this study are available from the corresponding author upon reasonable request.
